# Microbe-derived cyclopropane lipids activate host nuclear receptors

**DOI:** 10.1016/j.jlr.2025.100923

**Published:** 2025-10-06

**Authors:** William J. Massey, J. Mark Brown

**Affiliations:** 1 Department of Inflammation and Immunity, Cleveland Clinic, Cleveland, OH; 2 Center for Microbiome and Human Health, Cleveland Clinic, Cleveland, OH; 3 Department of Cancer Biology, Cleveland Clinic, Cleveland, OH

**Keywords:** FAs, microbiome, PPAR, metabolism

The gut microbiome has come into focus in biomedical research over the last 2 decades, given strong associations with cardiometabolic disease. While most studies largely characterize the community structure of microbiomes in health and disease contexts using sequencing-based approaches, a growing number of studies are going beyond sequencing-based characterization of “who” is there. Instead, many studies are focused on functional outputs derived from microbial metabolism of dietary substrates that liberate unique metabolites originating solely from bacteria including lipids. Activity in this emerging area of lipid research is aimed at identifying the molecular pathways through which these xenometabolites exert their physiological effects on the human host. One of the first documented examples of bacterially derived lipids impacting human health was the discovery of isomers of conjugated linoleic acids (CLAs), which were initially characterized in the 1990s as anticarcinogenic and antiobesity compounds originating from ruminant bacteria (1). These products of microbial transisomerization of dietary PUFA metabolism were shown to affect host physiology through the nuclear receptors, PPARs and liver X receptor alpha (2). This was the first example of how bacterially derived lipids can exert profound effects on host physiology and disease, but since the seminal discoveries with CLA isomers, a number of other examples have emerged that open the possibility that bacterial lipids may in some cases hold untapped therapeutic potential. However, our understanding of diet-microbe-host interaction in lipid metabolism is still in its infancy.

In one such example recently published in the *Journal of Lipid Research*, Debédat *et al.* (3) sought to understand whether microbial-derived cyclopropane FAs (CpFAs) engage with PPARs in a similar way to MUFAs that are known to activate these host nuclear receptors. Although there is very little known in regard to microbe-host interactions involving CpFAs, there is a wealth of knowledge on the role that CpFAs play in bacteria. CpFAs were first described in 1962 and shown to be quite abundant in a large number of diverse bacteria (4). Production of CpFAs occurs through cyclopropane FA synthases that add the methylene carbon from S-adenosylmethionine across the *cis* double bond of an MUFA incorporated into a phospholipid. These FAs play an important role in microbial membranes by conferring increased resistance to environmental stressors, which have implications for fitness and pathogenicity (5). While there has been ample research investigating the role of CpFAs in bacterial phospholipids, more recently, studies have been carried out that describe the presence of these FAs in food and their distribution throughout the mammalian body (6), putting forth the idea that these microbial FAs may impact host health and disease. Previously, *cis*-9,10 methylenehexadecanoic acid, a CpFA (16Δ1 *cis*-9) derived from palmitoleic acid (16:1 *cis*-9), had been identified as an activator of human adhesion G-protein-coupled receptor B1 (7). The important work by Debédat *et al.* (3) expands upon this concept by investigating broadly CpFA interactions with PPARs.

In humans, there are three PPAR genes (*NR1C1*, *NR1C2*, and *NR1C3* encoding PPARα, PPARδ, and PPARγ proteins, respectively) that are a clinically relevant class of nuclear hormone receptors relevant to many diseases. FA derivatives are well-known ligands for PPARs, and ligand-dependent activation results in reorganization of lipid synthetic and catabolic processes that help maintain organ and systemic lipid homeostasis. In fact, there are multiple Food and Drug Administration-approved drugs and many preclinical compounds that modulate PPAR signaling, thereby underscoring the potential importance of PPAR engagement by endogenous ligands as well as xenobiotic compounds. Notable disease states that are well positioned to be investigated following this line of work are type 2 diabetes mellitus and metabolic dysfunction-associated steatotic liver disease, which are managed with PPAR-targeting drugs but at the cost of multiple undesired side effects (8). Beyond these metabolic diseases already treated by PPAR agonists, there is also an interest in modulating PPAR signaling in diseases, such as cancer and inflammatory bowel disease (8). Given the key role that PPARs play in metabolic homeostasis, there is resurgent interest in finding new ways to selectively agonize each receptor for maximal benefit to human health.

Critical to the investigation by Debédat *et al.* (3) was the use of modern computational tools that allowed them to thoroughly characterize theoretical as well as known CpFA species, which enabled them to better understand the role of chain length and position of cyclopropanation. After these computational studies, the authors were able to show binding of CpFAs to PPARs (especially PPARα and PPARδ) in vitro using commercially available time-resolved fluorescence resonance energy transfer assays. Additionally, the authors sought to demonstrate in vivo activation of PPARs (especially PPARδ and PPARγ), which was accomplished by measuring gene expression of *Angptl4*, a known pan-PPAR target gene, in mouse 3T3-L1 cells treated with CpFAs in the presence or the absence of PPAR-specific antagonists. Collectively, the studies by Debédat *et al.* demonstrate the powerful potential of leveraging computer modeling to inform subsequent experiments, and further optimization of these workflows will advance the numerous biological fields of study. By combining computational modeling with experimental validation, this body of work has uncovered an important new microbe-host signaling axis that may be active under physiological conditions.

Although a vastly understudied area in lipid research, there are a few examples of other related gut microbe-derived FA metabolites having striking effects on host lipid metabolism and cardiometabolic disease. One important study showed that several *Bifidobacterium* and *Lactobacillus* species encode enzymes called linoleate oxidoreductase (CLA-DH) and linoleate hydratase (CLA-HY) that convert dietary linoleic acid (LA = 18:2, w-6) to LA derivatives in the CLA family including 10-hydroxy-*cis*-12-octadecenoic acid (9). 10-Hydroxy-*cis*-12-octadecenoic acid and related bacterially derived CLA metabolites were shown to engage host G-protein-coupled receptors (GPR40 and GRP120) to reduce inflammation and adiposity in mice (16). Similarly, gut microbe-derived metabolites of a-linolenic acid (18:3, w-3), broadly referred to as “conjugated linolenic acids,” were recently shown to suppress inflammation and limit oxidative stress in the host (10). Another recent example of metaorganismal lipid metabolism showed that the bacterially derived γ-linolenic acid (18:3, w-6) metabolite 10-oxo-*cis*-6,*trans*-11-octadecadienoic acid (γ-KetoC) acts as an anti-inflammatory signal in the gut (11). It is important to note that the bacterial genes responsible for the production of these diverse bacterial PUFA metabolites are common among human microbiomes and are associated with variability in susceptibility to cardiometabolic disease (12). Of therapeutic interest, CLA and conjugated linolenic acid isomers have very striking antiobesity, antiatherosclerotic, and anticancer effects (2, 10, 11, 12) in mice and humans, providing proof of concept that bacterial-derived lipids can powerfully reorganize host lipid metabolism and hold untapped therapeutic promise.

Although this recent study by Debédat *et al.* (3) brings to light the potential mechanisms through which CpFAs may exert their effects on human health and disease, more work is needed to experimentally validate these exciting findings in human systems and in animal models of disease. Further, there is a need to understand the events upstream of signaling such as the regulation of CpFA production by the gut microbiome in response to substrate availability and environmental stressors as well as the host trafficking and metabolism of CpFAs, which will affect signaling. It will be important to develop quantitative analytical assays to determine whether circulating or tissue levels of CpFAs are altered in diverse cardiometabolic diseases or cancers. This endeavor will require substantial synthetic chemistry capabilities because standards are not currently available. Due to the fact that CpFAs are isobaric (with identical elemental composition) to MUFAs of one-carbon longer chain length, native and stable isotope-labeled standards for the many diverse molecular species of CpFA will be needed for quantitative assays. It remains possible that the position of cyclopropane ring on FA backbone can dramatically alter sensing by host nuclear receptors. Furthermore, it will be important to understand whether the role that CpFAs play in bacterial stress responses may intersect with host antimicrobial or metabolic responses after dietary substrates are consumed. It is also very likely that microbe-derived CpFAs can be esterified or metabolized in unanticipated ways by host lipid metabolic enzymes to channel these bacterial lipid metabolites into the complex lipidome in the human metaorganism. If CpFAs are assimilated in host lipids, there are unanswered questions with regard to how they are packaged in lipoproteins, complexed to albumin, metabolized, and transported from the gut to the liver and peripheral tissues. Given structurally similar diet- and host-derived FAs lacking the cyclopropane rings are used for metabolic fuel, building blocks for cell membranes, and signaling molecules, it will be important to further understand how CpFAs may compete with or alter normal FA metabolism. Although the field of lipid biochemistry and physiology has made tremendous strides in understanding how abnormal lipid metabolism in human cells contributes to many human diseases, the work by Debédat *et al.* (3) reminds us that bacterial lipid metabolism may have unexpected roles in shaping the lipidome in the human metaorganism. Importantly, advancing our understanding in diet-microbe-host interactions in lipid metabolism has tremendous potential to uncover new therapeutic targets. There is a fertile period that lies ahead in lipid research where instead of only targeting host lipid metabolic enzyme or receptors, we instead selectively target the gut microbial production of lipid metabolites such as CpFAs and many others to impact human health.

## Conflict of interest

The authors declare that they have no conflicts of interest with the contents of this article.
